# Hepatitis B Virus Prevalence among HIV-Uninfected People Living in Rural and Peri-Urban Areas in Botswana

**DOI:** 10.3390/microorganisms12061207

**Published:** 2024-06-15

**Authors:** Motswedi Anderson, Thabo Mangogola, Bonolo B. Phinius, Gorata Mpebe, Christopher O. Aimakhu, Wonderful T. Choga, Basetsana Phakedi, Lynnette N. Bhebhe, Doreen Ditshwanelo, Kabo Baruti, Linda Mpofu-Dobo, Lebogang Othusitse, Tsholofelo Ratsoma, Tendani Gaolathe, Joseph Makhema, Roger Shapiro, Shahin Lockman, Sikhulile Moyo, Simani Gaseitsiwe

**Affiliations:** 1 Botswana Harvard Health Partnership, Private Bag BO320, Gaborone, Botswana; manderson@bhp.org.bw (M.A.); mangogolat@gmail.com (T.M.); bphinius@mail.bhp.org.bw (B.B.P.); gmpebe@bhp.org.bw (G.M.); wchoga@bhp.org.bw (W.T.C.); bphakedi@bhp.org.bw (B.P.); lbhebhe@bhp.org.bw (L.N.B.); dditshwanelo@bhp.org.bw (D.D.); kbaruti@bhp.org.bw (K.B.); ldobo@bhp.org.bw (L.M.-D.); loapiothusitse@gmail.com (L.O.); tratsoma@bhp.org.bw (T.R.); gaolathet@gmail.com (T.G.); jmakhema@bhp.org.bw (J.M.); rshapiro999@gmail.com (R.S.); shahin.lockman@gmail.com (S.L.); smoyo@bhp.org.bw (S.M.); 2 Africa Health Research Institute, Durban 4013, South Africa; 3 The Francis Crick Institute, 1 Midland Road, London NW1 1AT, UK; 4 Pan-African University (Life and Earth Sciences Institute), University of Ibadan, Ibadan 200132, Nigeria; chrisaimakhu@yahoo.com; 5 School of Allied Health Professions, Faculty of Health Sciences, University of Botswana, Private Bag UB 0022, Gaborone, Botswana; 6 Department of Biological Sciences, Faculty of Science, University of Botswana, Private Bag UB 0022, Gaborone, Botswana; 7 Department of Biological Sciences and Biotechnology, Faculty of Sciences, Botswana International University of Science and Technology, Private Bag 16, Palapye, Botswana; 8 Faculty of Medicine, University of Botswana, Private Bag UB 0022, Gaborone, Botswana; 9 Department of Immunology and Infectious Diseases, Harvard T.H. Chan School of Public Health, Boston, MA 02115, USA; 10 Division of Infectious Diseases, Brigham and Women’s Hospital, Boston, MA 02115, USA; 11 Division of Medical Virology, Faculty of Medicine and Health Sciences, University of Stellenbosch, Stellenbosch, Private Bag X1, Matieland 7602, South Africa; 12 School of Health Systems and Public Health, University of Pretoria, Private Bag X20, Pretoria 0028, South Africa

**Keywords:** hepatitis B virus, HBV, prevalence, genotypes, mutations, HIV negative, Africa

## Abstract

(1) Background: we determined the prevalence of the hepatitis B virus (HBV) amongst people without human immunodeficiency virus (HIV) in rural and peri-urban areas in Botswana. (2) Methods: We screened for the hepatitis B surface antigen (HBsAg) from archived plasma samples of people without HIV (n = 2135) randomly selected from the Botswana Combination Prevention Program (BCPP) (2013–2018). We sequenced 415 bp of the surface region using BigDye sequencing chemistry. (3) Results: The median age of participants was 31 (IQR: 24–46) and 64% (1360/2135) were female. HBV prevalence was 4.0% (86/2135) [95% CI: 3.3–4.9]) and ranged between 0–9.2%. Older participants (>35 years) had increased odds of HBV positivity (OR: 1.94; 95% CI: [1.32–2.86]; *p* = 0.001). Thirteen samples were sequenced and seven (53.8%) were genotype A, three (23.1%) were genotype D and genotype E each. Clinically significant mutations were identified in the surface region, but no classic drug resistance mutations were identified. (4) Conclusions: We report an HBV prevalence of 4.0% (95% CI 3.3–4.9) among people without HIV in rural and peri-urban communities in Botswana with varying rates in different communities. A comprehensive national HBV program is required in Botswana to guide HBV prevention, testing and management.

## 1. Introduction

Approximately 254 million people worldwide are infected with chronic hepatitis B virus infections (CHB), with most cases prevalent in the Western Pacific region and sub-Saharan Africa, where 96.8 million and 64.7 million people are affected, respectively [1]. In Botswana, hepatitis B surface antigen (HBsAg) prevalence ranging between 1.1% and 10.6% has been reported in blood donors, pregnant women and mostly in people with human immunodeficiency virus (PWH) [2,3,4,5,6,7,8]. Most of these studies were in urban areas except for the largest HBV study in PWH in the country which was conducted in rural and peri-urban areas [4]. This study reported an overall prevalence of 8% with varying prevalence rates between communities some differing by as much as 10-fold (2.1–22.1%) [4]. Several studies reported similar HBV prevalence rates in PWH and those without, while others reported differences [7,9,10]. 

HBV is classified into 10 genotypes (A-I) and a putative genotype J based on a nucleotide divergency of >7.5% with more than 35 subgenotypes [11,12,13]. Genotypes differ by geographic distribution and by disease progression [13,14,15]. In Africa, including in Botswana, genotypes A, D and E have been reported [8,14,16]. Subgenotype A1 predominantly found in Africa has been linked with increased chronicity and faster progression to hepatocellular carcinoma [14,15]. 

One of the barriers to elimination, especially in Africa, is the lack of national data hence most of the national HBV prevalence rates have been extrapolated from the little data available within the region [10,17,18]. In Botswana, there is no prevalence data of HBV at the population level among people without HIV; therefore, we aimed to establish the HBV prevalence amongst people without HIV across rural and peri-urban areas in Botswana. 

## 2. Materials and Methods

### 2.1. Study Population

Archived plasma samples from people without HIV who were enrolled in the Botswana Combination Prevention Project (BCPP) were used. BCPP was a pair-matched, cluster-randomized trial conducted in 30 communities (15 pairs matched according to size, pre-existing health services, population age structure and geographical location, including proximity to urban areas) around Botswana from 30 October 2013 to 24 November 2018 [19]. BCPP enrolled 12,610 participants aged 16–64 years, of whom 9014 were without HIV [20]. A total of 2135 samples from people without HIV were randomly selected for characterization in this study ensuring proportional distributions amongst the communities. Out of 30 BCPP communities, 2 were not sampled (Otse and Molapowabojang) because there were no stored samples available for these sites. The participants distribution per community is shown in Table 1 below. Samples were stored at −80 °C prior to testing.

### 2.2. Ethical Approval

Approval for the study was sought from the Institute of Advanced Medical Research and Training (IAMRAT) approval number UI/EC/22/0001 and the Human Research Development Committee (HRDC) at the Botswana Ministry of Health (MoH), HRDC number 01028. Participants provided written informed consent for their samples to be used in the parent study as well as subsequent studies.

### 2.3. Serological Assays

#### 2.3.1. Validation of the HBsAg Dilution Protocol

Plasma samples had insufficient volumes, so they were diluted using the previously validated in-house protocol shown below. Briefly, archived plasma samples from people with HIV (PWH) from the Botswana Combination Prevention Project (BCPP) were used for the validation of the HBsAg dilution protocol. Samples from PWH were used because they had enough sample volume to allow for the validation. Twenty-four HBsAg positive samples with known optical densities (ODs) were used. The samples were divided into three categories: high positive (OD > 4.9, n = 6), medium positives (OD range 2.0–4.9, n = 9) and low positives (OD range 0–1.9, n = 9) based on their ODs. Serial dilutions of 1:10, 1:100, 1:1000 and 1:100,000 of sample: phosphate buffered saline (PBS) were performed. HBsAg was then screened in the samples with the various dilutions using the Murex HBsAg Version 3 enzyme-linked immunosorbent assay (ELISA) kit (Murex Biotech, Dartford, UK) following manufacturer’s instructions. The results showed that all high and medium positives remain positive from the 1:10 to the 1:10,000 dilutions. The low positives were all positive at the 1:10 dilution; however, (4/9) 44% of low positives lost HBsAg at the 1:100 dilution and an additional (3/9) 33% lost the HBsAg positivity by the 1:1000 dilution. These results show that regardless of the OD, 10^−1^ dilution remains positive.

#### 2.3.2. HBsAg Screening

Plasma samples had insufficient volumes, so they were diluted using the previously validated in-house protocol shown above. Briefly, prior to HBsAg screening, samples were diluted using PBS at a 10-fold dilution which detects 100% of samples with positive HBsAg serology (HBsAg+) including those with low optical densities. After dilution, the samples were screened for HBsAg using a Murex HBsAg Version 3 enzyme-linked immunosorbent assay (ELISA) kit (Murex Biotech, Dartford, UK). HBsAg+ samples were subsequently repeated for confirmation following the manufacturer’s guidelines. 

### 2.4. Amplification and Sequencing

Total nucleic acid was extracted from 200 μL of HBsAg+ plasma samples diluted with PBS at 10-fold using an in-house validated protocol. We used the QIAamp DNA Blood (QIAGEN, Hilden, Germany) following the manufacturer’s instructions with an elution volume of 30 μL [21]. A 415 bp fragment of the surface/polymerase gene was amplified by semi-nested PCR with two rounds of amplification using Platinum Taq DNA Polymerase High Fidelity kit according to the manufacturer (Invitrogen, Waltham, MA, USA), using primers and thermal cycling conditions as described previously [16,22]. Sequencing was performed using big dye sequencing chemistry. Briefly, the master mix for sequencing PCR consisted of 3 µL Sequencing Buffer (5×), 1 µL BigDye^®^Terminator v3.1, 0.2 µL of 2 µM Primer, 1 µL purified PCR product and 4.8 µL in a 10 µL reaction mix. The cycling conditions were 5 min denaturing at 94 °C, and then 30 cycles of denaturing for 45 s at 94 °C, annealing for 30 s at 50 °C, and elongation at 72 °C for 90 s, with extension at 72 °C for 10 min using HBV840 (5′-GTTTAAATGTATACCCAAAGAC-3′; nt840–861) and HBV381 (5′-TGCGGCGTTTTATCATCTTCCT-3′; nt381–402) primers for first round. Second round cycling conditions were denaturation at 94 °C for 5 min and then 30 cycles of denaturation at 94 °C for 45 s, annealing at 55 °C for 30 s, elongation at 72 °C for 60 s, and extension at 72 °C for 10 min using HBV381 and HBV801 (5′-CAGCGGCATAAAGGGACTCAAG-3′ nt801–822) primers. Sequencing reactions were set up using big dye sequencing chemistry and two primers: HBV 381 and HBV 801. ZR DNA Sequencing Clean-up Kit (Zymo, Irvine, CA, USA) was used for sequencing clean-up according to the manufacturer’s protocol. Sanger sequencing was performed on an ABI 3130xl genetic analyzer (Applied Biosystems, Foster City, CA, USA).

### 2.5. Data Analyses

Manual editing of sequences were carried out using Sequencher v5.0 software (Gene Codes Corp., Ann Arbor, MI, USA). Consensus sequences were generated from the overlapping sequences. Next, consensus sequences generated were viewed, trimmed and aligned with all previously generated Botswana reference sequences from GenBank using AliView alignment viewer version 1.28 [23]. Sequences were then uploaded to the Geno2Pheno online database (http://www.geno2Pheno.org, accessed on 4 April 2024) for genotypic, drug resistance and escape mutations analyses. Sequences were further uploaded to a second online database, the Stanford HBVseq database, (http://hivdb.stanford.edu/HBV/HBVseq/development/HBVseq.html, accessed on 4 April 2024) to confirm genotype and drug resistance mutations. We constructed a maximum-likelihood tree using the best fitting model of nucleotide substitution [TVMe+R2] using IQTREE with 1000 bootstrap replicates [24].

### 2.6. Statistical Analysis

Predictors associated with HBV positivity were determined using both univariate and multivariable logistic regression, adjusting for clustering by community. Pairwise comparison of median age by community and between HBV negative and positive cases in each community was conducted using Wilcoxon rank-sum test. Kruskal–Wallis test was used to compare the median age across the different communities. To adjust for multi-comparison between communities, the Dunn’s test with Bonferroni correction was used.

Data visualization was performed in R version 4.3.0. Stata version 14.0 (Stata Corp., College Station, TX, USA) was used for statistical analysis and *p*-values < 0.05 were considered statistically significant. 

## 3. Results

### 3.1. HBV Prevalence (HBsAg Positivity)

The median age of all participants was 31 (IQR: 24–46) and of these, 63.7% (1360/2135) [95% CI: 61.6–65.7] were female. We report an overall HBV prevalence of 4.0% (86/2135) [95% CI: 3.3–4.9]. Univariate logistic regression analysis shows that participants aged 35 years and older have increased odds of HBV positivity (OR: 1.94; 95% CI: [1.32–2.86]; *p* = 0.001) (Table 2, Supplementary Figure S1). We observed reduced odds of HBV positivity among people with secondary education (OR: 0.37; 95% CI: [0.17–0.81]; *p* = 0.012) and those with higher education levels (OR: 0.35; 95% CI: [0.14–0.88 *p* = 0.025) compared to those with non-formal education. Married participants had increased odds of HBV positivity (OR: 1.67; 95% CI: [1.15–2.41]; *p* = 0.007) and were older (Table 2, Supplementary Figure S2). There was a trend towards more inconsistent condom use in the HBV positive group compared to the HBV negative group. There were no significant independent predictors of HBV as shown in the multivariate logistic regression in Table 2.

**Figure 1:**
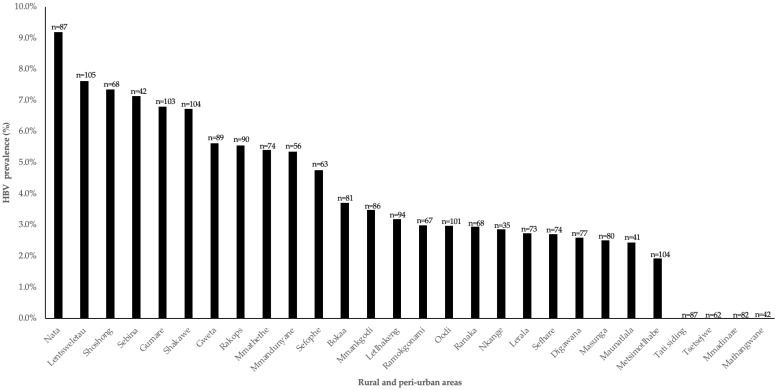
HBV prevalence by community among people without HIV from the BCPP cohort.

HBsAg HBV prevalence was analysed for the 30 BCPP villages. HBV prevalence r ranged between 0–9.2% with Nata having the highest HBV prevalence among people without HIV, while Mmadinare, Mathangwane, Tsetsejwe and Tati siding reported no HBVsAg positive cases. Figure 1.

Among participants who were older (≥35 years), HBV prevalence in males was 7.8% vs. 4.6% in females, although not statistically significant (*p* = 0.06), (Supplemental Figure S3, Table 2).

### 3.2. Age Distribution amongst the Communities

**Figure 2:**
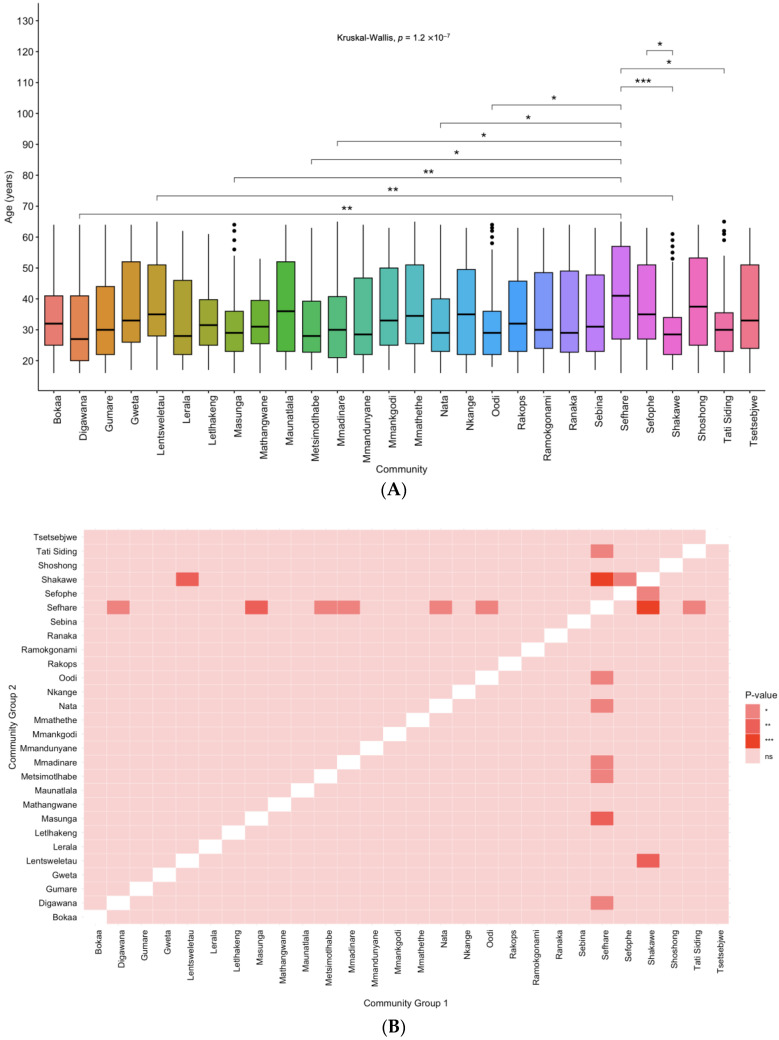

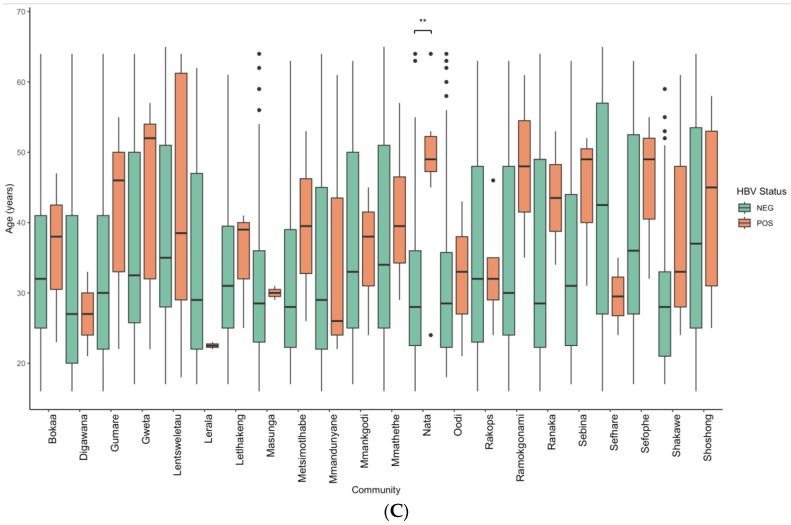
(**A**) Age by community, (**B**) Heat map showing differences in age distribution between communities, (**C**) Boxplots showing the age distribution across the BCPP communities with both HBV negative (green) and positive (orange) cases. Pairwise comparison was conducted by age between communities and between HBV negative and positive cases using Wilcoxon test. The significance levels of ns, *, **, and *** represent *p*-values > 0.05, <0.05, <0.01 and <0.001, respectively.

The differences in age by community were determined. There was a significant difference in age by community (Figure 2A,B). There was no significant difference in age between the community with the highest HBV prevalence (Nata), versus those with no HBV positive cases reported (Mmadinare, Mathangwane, Tsetsejwe and Tati siding) (Figure 2B). The differences in HBV status by age in each community was also determined. Four communities without any HBV cases, namely Mmadinare, Mathangwane, Tsetsebjwe and Tati siding, and two communities namely Maunatlala and Nkange with only 1 HBV positive case each, were excluded from the analysis. Overall, HBV positive participants tended to be older as shown in Figure 2C and this difference was statistically significant for Nata (*p* = 0.001). 

### 3.3. Prevalence by Vaccination Status

There were no HBV vaccination records for the study participants; hence, participants who were ≤21 years were classified as likely to be vaccinated based on the implementation of HBV infant vaccination in Botswana in 2000. The HBV prevalence in the likely vaccinated group was 0.8% (3/363) [95% CI: 0.3–2.4]. The HBV prevalence in the likely unvaccinated group (≥22 years) was 4.7% (83/1772) [95% CI: 3.8–5.8]. There was a statistically significant difference in HBV status between the HBV vaccinated group versus the unvaccinated group, *p* value = 0.0007.

### 3.4. HBV Genetic Diversity 

**Figure 3:**
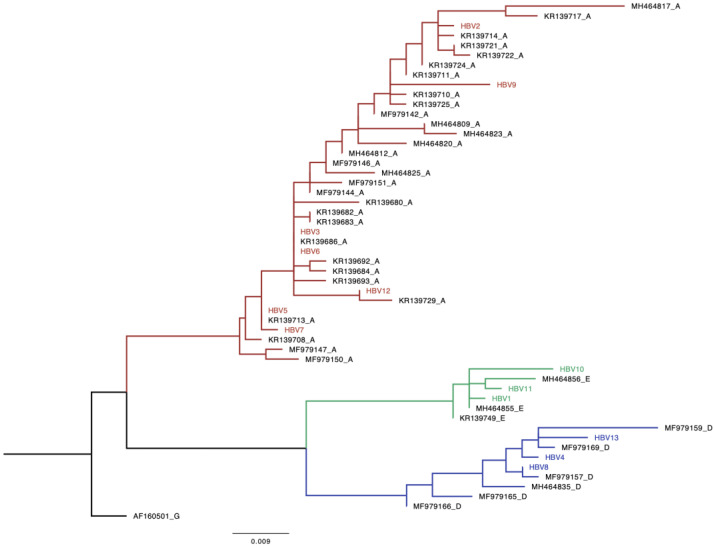
Phylogenetic analysis of HBV strains obtained from HIV-uninfected participants living in rural and peri-urban areas in Botswana. A phylogenetic analysis of a 415 bp fragment of the HBV from different isolates with seven HBV A, three HBV D and three HBV E sequences clustered with the matching HBV genotype Botswana reference. Study sequences are denoted by the name HBV followed by a number and the references begin with an accession number followed by a letter denoting the genotype. Genotype A is coloured red, D is coloured blue and E is coloured green. Black is genotype G which was used for rooting.

Out of the 86 HBV^+^ samples, n = 13 (15%) were amplified and all of them (100%) were successfully sequenced. The online databases and the phylogenetic analysis revealed that HBV genotype A, n = 7 (54%), was found to be the most predominant circulating HBV genotype while the remaining six sequences were equally shared between genotypes D (23%) and E (23%). The 13 newly generated sequences were used to construct a maximum-likelihood tree and the available online sequences from Botswana were used to confirm genotypes and determine clustering patterns of these sequences with reference sequences. The tree shows that the sequences generated from our study clustered with reference sequences from Botswana (Figure 3).

**Figure 4:**
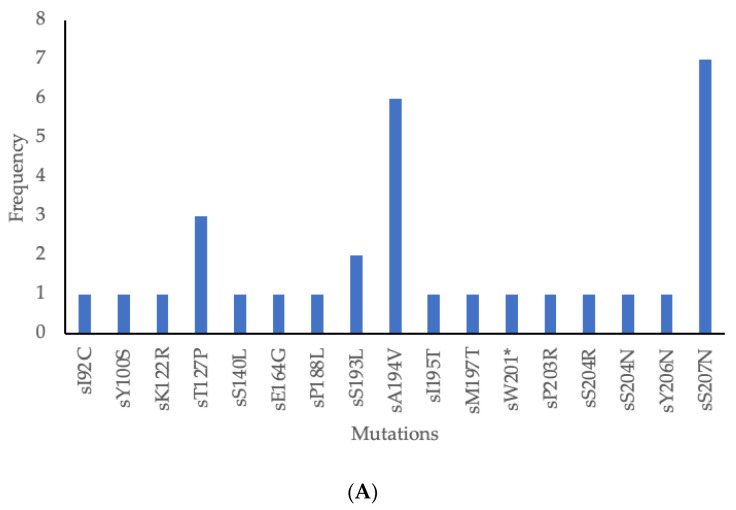

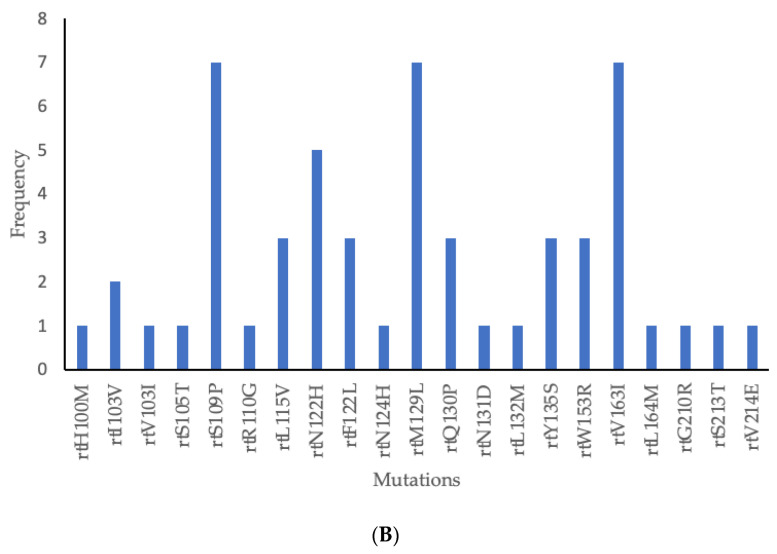
(**A**) Frequency of surface region mutations, (**B**) Frequency of rt region mutations. *: stop codon.

The 415 bp fragment covered aa 82–210 of the surface region which includes the major hydrophilic region (MHR) (aa 99–169). As depicted in Table 3 below, 38 mutations were found in 12/13 isolates (92.3%) showing amino acid substitution in the HBV surface (s) gene and the overlapping reverse transcriptase (rt) region. Amongst these, 17 were in the s region and 21 in the rt region. Only two mutations were novel. Clinically significant mutations included escape mutations as well as those associated with disease progression and OBI were identified in this study (Table 4). The most common mutations in the s region were sS207N and sA194V found in seven (54%) and six (46%) participants, respectively, (Figure 4A). The most common mutations in the rt region were rtS109P, rtM129L and rtV163I found in seven (54%) each. These three mutations appeared together in six participants (Figure 4B).

## 4. Discussion

To our knowledge, the current study is the largest study to report HBV prevalence in people without HIV in rural and peri-urban communities in Botswana. The relationship between the prevalence of HBV and participant socioeconomic demographics was explored. We report a 4.0% HBV prevalence which is higher than approximately 1% previously reported in pregnant women without HIV in Botswana and South Africa but similar to a serosurvey in South Africa [3,10,41]. The differences in these prevalence rates might be due to differences in geographic regions, cultural practices, access to healthcare and vaccination coverage as the study on pregnant women was conducted in urban areas while the current study was in peri-urban and rural areas. Differences in HBV prevalence rates even in the same country have been reported before including in Botswana [4,42]. Notably, this prevalence is lower than that reported in PWH in the same communities which was 8% overall [4]. Higher rates of HBV prevalence in PWH compared with people without HIV have been reported before in South Africa [10] while some studies, including a multicentre study which included participants from Botswana, South Africa, Kenya, Malawi, India, Thailand and Brazil, reported similar rates [3,7,43]. The differences might be due to differences in risk factors found in different places as well as the level of immunosuppression in the HIV infected groups. HIV associated immunosuppression has been associated with HBV reactivation resulting in a higher HBsAg positivity [44,45]. In Africa, HBV infections mostly occur during early childhood due to horizontal transmissions while HIV infections are mostly due to sexual transmissions. However, owing to some sexually transmitted cases of HBV in adulthood, and HBV reactivations associated with immunodeficiencies emanating from HIV infection, some studies have found a higher HBV prevalence in PWH. There was a lower HBV prevalence in the likely vaccinated group compared to the unvaccinated group as expected. HBV infant vaccination commenced around 2000 in Botswana including the birth dose [6].

In this study, there were varying HBV prevalence rates across communities, with the highest prevalence recorded in Nata (9.2%) while some places such as Tati, Tsetsebjwe, Mmadinare and Mathangwane reported no HBV cases. Differences in prevalence between communities have been reported before including in PWH in the same communities [4]. Interestingly, the HBV prevalence spread is different between PWH and people without HIV in these communities [4]. For example, Nata had the highest HBV prevalence in people without HIV whereas it had the ninth highest prevalence out of thirty communities in a study on HBV prevalence in PWH. In our current study, Tati and Tsetsebjwe also reported no cases of HBV in people without HIV but had the third and twelfth highest HBV prevalence in the same study of PWH, respectively [4]. There was a difference in age amongst the communities and HBV positive cases were older, particularly in Nata; notably this is the community with the highest HBV prevalence. There was a difference in age amongst the communities. Interestingly, this might not explain the differences in prevalence rates. For example, there was no significant age difference between the community with the highest HBV prevalence and all four communities with no HBV positive cases. Interestingly, HBV positive participants tended to be older, but the difference was statistically only significant for Nata. Although we noticed these differences by age, the HBV rates did not differ by age within the community except in Nata. The differences between the places might be due to cultural practices. It is also possible that the current study randomly selected HBV negative participants as only a subset of samples was screened for HBV.

Older age, marriage and secondary education were predictors of HBV positivity in univariate analysis but there were no predictors of HBV positivity in multivariate analysis. In this study, the older age group are more likely to be unvaccinated as infant vaccination commenced around 2000 when the current study participants were at least adolescents [6]. Married people were also older and hence were likely to be unvaccinated leading to increased odds of HBV positivity. Other studies showed no association with education but showed an association with age [10,44]. The South African study also did not show relationship status as well as the number of lifetime partners as predictors of HBV positivity similar to the current study [10]. Most studies reported more HBV in males; however, for the current study, sex was not a predictor of HBV positivity [4,44,46,47]. More HBV positivity in males has been linked to the effective immune system in females [47]. In a study in PWH in the same communities, the male sex and northern geographic region were independent predictors of HBV positivity unlike in the current study. In the said study with PWH, being older was only a predictor in univariate analysis similar to the current study [4]. Predictors of HBV vary across studies which might be due to lifestyle practices, genotypes as well as host genetics [10,47]. 

The distribution of the genotypes across peri-urban and rural areas around Botswana will aid in furthering our understanding of the prevalent HBV genotypes amongst people without HIV. Similar to the existing literature on the HBV genotypes circulating in sub-Saharan Africa, the HBV genotypes A (54%), D (23%) and E (23%) were also found to be prevalent in Botswana [8,21]. Genotypes A, D and E from this study clustered together with sequences of known HBV genotypes from Botswana suggesting local transmission. Information on the distribution of HBV genotypes across rural and peri-urban areas in Botswana is limited. 

This study identified mutations, some of which were of clinical significance. In the surface region, sA194V and sS207N were the predominant mutations as reported in a study from Kenya [48]. A194V has not been characterized but it has been reported in many studies while sS207N is linked to immune escape [38,39,40]. Other mutations identified which were associated with immune escape were sK122R, sT127P and sS140L which are found in the MHR [28,29,30,31,32,33]. Other mutations with clinical significance were sY100S, sK122R, sM197T, sW201*, sS204R and sS204N which impair the production and secretion of HBsAg, associated with low HBV viral load and OBI [25,26,27,28,29,34,35,37]. In the polymerase region, the most common mutations were rtS109P, rtM129L and rtV163I found in seven participants each similar to other studies which reported these mutations in genotype A and indeed these mutations were present in genotype A in this study [49,50,51]. These three mutations appeared together in six genotype A participants; rtS109P was also found in genotype E. We report no classic drug resistance-associated mutations such as rtL80I/V, rtV173L, rtL180M, rtA181S, rtA194T, rtS202I, rtM204V/I, rtN236T and rtM250L/V associated with resistance against lamivudine, telbivudine, adefovir, entecavir and tenofovir in this study similar to other studies [21,51,52]. The rtY135S mutation was mostly seen in the treatment of non-responders and some studies classified it under drug resistance mutations against lamivudine, telbivudine, adefovir and entecavir but its characterization is not yet concluded [53,54,55,56]. This contrasts with a study in PWH in the same communities which reported a high prevalence of drug resistant mutations against lamivudine [57]. These results might suggest that there were no drug resistant mutations transmitted between PWH and those without HIV. We identified uncharacterized mutations which have been reported in other studies (sE164G, sP188L, sS193L, sA194V, sI195T, sY206N, rtI103V, rtV103I, rtS105T, rtS109P, rtR110G, rtL115V (only found in genotype D), rtN122H, rtF122L, rtN124H, rtQ130P, rtN131D, rtL132M, rtW153R, rtV163I, rtL164M, rtS213T, rtG210R and rtV214E) [21,49,50,54,58,59,60,61,62,63,64,65,66,67,68,69,70]. We also reported novel mutations in this study, sI92C and rtH100M, all found in. genotype E. 

This study bears the strength of being the largest study on HBV in people without HIV in understudied rural and peri-urban areas in Botswana. The limitations of the study include insufficient sample volumes which necessitated the use of a dilution protocol which however can be applied in future studies. The effects of insufficient sample volumes were that we could not test for more HBV biomarkers such as HBV surface antibodies and the samples were diluted which might have affected the sequencing success, which was low in our study. The small number of sequences limits genotype distribution and mutations. The community level HBV prevalence should be taken with caution as the study was not powered to determine the prevalence at community level. The small number of samples tested per community limit HBV prevalence comparisons by community. There were no HBV vaccination records and introduction of infant vaccination in Botswana was used to infer vaccination status.

## 5. Conclusions

We report an HBV prevalence of 4.0% among people without HIV in rural and peri-urban communities in Botswana with varying rates in different communities and the presence of clinically relevant mutations. Furthermore, the HBV prevalence was lower in the likely vaccinated group indicating the success of the vaccination program. Integration of general care, management and advocacy in HBV regardless of HIV status is urgently needed in Botswana to accelerate reductions in the prevalence of HBV among the general population especially in the unvaccinated group. Surveillance of clinically relevant mutations is recommended to guide prevention strategies. Future studies screening for further HBV biomarkers are needed to further characterize HBV in people without HIV in Botswana.

## Figures and Tables

**Table 1:** The distribution of participants per community.

Village	Total Number of Participants	Tested (n = 2135)
Metsimotlhabe	394	104
Molapowabojang	344	0
Oodi	377	101
Digawana	189	77
Bokaa	366	81
Otse	377	0
Letlhakeng	411	94
Gumare	399	103
Rakops	362	90
Mmankgodi	349	86
Lentsweletau	308	105
Gweta	356	89
Shoshong	329	68
Tati Siding	341	87
Shakawe	379	104
Ranaka	135	68
Sebina	190	42
Mmadunyane	222	56
Masunga	335	80
Nata	337	87
Sefhare	275	74
Mmathethe	277	74
Ramokgonami	244	67
Mmadinare	301	82
Mathwangwane	254	42
Sefophe	296	63
Lerala	317	73
Nkange	114	35
Tsetsebjwe	229	62
Maunatlala	167	41

**Table 2:** Univariate and multivariate analysis of demographic factors associated with HBV positivity.

Characteristic	HBV+ (n = 86)	HBV− (n = 2049)	Univariate Logistic Regression		Multivariate Logistic Regression	
			OR (95% CI)	*p*-Value	aOR (95% CI)	*p*-Value
Sex, n (%)						
Female	49 (57.0)	1311 (64.0)	Ref		Ref	
Male	37 (43.0)	739 (36.0)	1.34 (0.73–2.46)	0.34	1.41 (0.77–2.58)	0.27
Age category, n (%)						
<35	38 (44.2)	1242 (60.6)	Ref		Ref	
≥35	48 (55.8)	807 (39.4)	1.94 (1.32–2.86)	0.001	1.40 (1.76–2.58)	0.28
Marital status, n (%)						
Single or never married	58 (67.4)	1557 (76.0)	Ref		Ref	
Married	26 (30.2)	419 (20.5)	1.67 (1.15–2.41)	0.007	1.15 (0.68–1.92)	0.61
Widowed/Divorced/separated	2 (2.3)	73 (3.5)	0.74 (0.9–2.87)	0.66	0.43 (0.10–1.82)	0.25
Region, n (%)						
South	30 (34.9)	760 (37.1)	Ref			
Central	23 (26.7)	639 (31.2)	0.91 (0.51–1.62)	0.75		
North	33 (38.4)	650 (31.7)	1.29 (0.74–2.24)	0.38		
Number of lifetime sexual partners (n = 1952)						
<10	68 (82.9)	1608 (86.0)	Ref			
≥10	14 (17.1)	262 (14.0)	1.26 (0.64–2.48)	0.50		
Education level, n (%)						
Non-formal	19 (22.1)	216 (10.6)	Ref		Ref	
Primary	19 (22.1)	335 (16.4)	0.64 (0.29–1.42)	0.28	0.64 (0.28–1.46)	0.29
Secondary	37 (43.0)	1133 (55.5)	0.37 (0.17–0.81)	0.012	0.45 (0.16–1.22)	0.12
Higher than senior secondary	11 (12.8)	359 (17.5)	0.35 (0.14–0.88)	0.025	0.41 (0.14–1.17)	0.10
Employment						
Unemployed	54 (62.8)	1444 (70.5)	Ref			
Employed	32 (37.2)	605 (29.5)	1.41(0.88–2.31)	0.17		
Circumcised (n = 775)						
No	29 (78.4)	495 (67.1)	Ref			
Yes	8 (21.6)	241 (32.7)	0.57 (0.29–1.12)	0.10		
Inconsistent condom use (n = 1632)						
Yes	48 (73.9)	943 (60.2)	Ref			
No	17 (26.2)	624 (39.8)	0.54 (0.28–1.04)	0.06		
Age at First Sex						
<18	22 (31.9)	487 (29.7)	Ref			
≥18	47 (68.1)	1155 (70.34)	0.90 (0.58–1.40)	0.64		

Abbreviations: HBV; hepatitis B virus, OR; Odds ratio, aOR; adjusted Odds ratio, HBV+; hepatitis B virus positive, HBV−; hepatitis B virus negative.

**Table 3:** HBV mutations.

Sample ID	Genotype	Substitution rt	Substitution S
MA1	E		
MA2	A	rtS109P, rtM129L, rtW153R, rtV163I	sS193L, sS207N
MA3	A	rtS109P, rtN122H, rtM129L, rtV163I	sA194V, sS207N
MA4	D	rtL115V, rtF122L, rtQ130P, rtY135S, rtG210R	sT127P, sW201 *
MA5	A	rtI103V, rtS109P, rtN122H, rtM129L, rtW153R, rtV163I	sA194V, sS207N
MA6	A	rtS109P, rtN122H, rtM129L, rtV163I	sA194V, sS207N
MA7	A	rtI103V, rtS109P, rtN122H, rtM129L, rtW153R, rtV163I	sA194V, sP203R, sS207N
MA8	D	rtV103I, rtL115V, rtF122L, rtQ130P, rtY135S, rtS213T	sT127P, sS204R
MA9	A	rtS105T, rtR110G, rtM129L, rtV163I	sS193L, sA194V, sI195T, sM197T, sS204N, Ss207N
MA10	E	**rtH100M**, rtS109P	**sI92C**, sS140L
MA11	E	rtL132M	sP188L
MA12	A	rtS109P, rtN122H, rtN124H, rtM129L, rtN131D, rtV163I	sK122R, sA194V, sS207N
MA13	D	rtL115V, rtF122L, rtQ130P, rtY135S, rtL164M, rtV214E	sY100S, sT127P, sE164G, sY206N

Abbreviation: rt: reverse transcriptase, s: surface. Bold = novel, *: stop codon.

**Table 4:** Clinical significance of identified HBV mutations.

Mutation	Genotype	Clinical Significance
**rtH100M**	E	Not characterized
rtI103V	A	Not characterized
rtV103I	D	Not characterized
rtS105T	A	Not characterized
rtS109P	A, E	Not characterized
rtR110G	A	Not characterized
rtL115V	D	Not characterized
rtN122H	A	Not characterized
rtF122L	D	Not characterized
rtN124H	A	Not characterized
rtM129L	A	Not characterized
rtQ130P	D	Not characterized
rtN131D	A	Not characterized
rtL132M	E	Not characterized
rtY135S	D	Partly linked to drug resistance
rtW153R	A	Not characterized
rtV163I	A	Not characterized
rtL164M	D	Not characterized
rtS213T	D	Not characterized
rtG210R	D	Not characterized
rtV214E	D	Not characterized
**sI92C**	E	Not characterized
sY100S	D	Impair production and secretion of HBsAg, associated with OBI [25,26,27]
sK122R	A	Immune escape, Associated with OBI [28,29,30]
sT127P	D	Immune escape [31,32]
sS140L	E	Immune escape [33]
sE164G	D	Not characterized
sP188L	E	Not characterized
sS193L	A	Not characterized
sA194V	A	Not characterized
sI195T	A	Not characterized
sM197T	A	Correlate with low HBV viral load [34]
sW201 *	D	Reduces intracellular HBsAg [35]
sP203R	A	Associated with HCC [36]
sS204R	D	Reduces HBsAg secretion [37]
sS204N	A	Correlate with low HBV viral load [34]
sY206N	D	Not characterized
sS207N	A	Immune escape [38,39,40]

Abbreviation: rt: reverse transcriptase, s: surface. Bold = novel, *: stop codon.

## Data Availability

The raw data supporting the conclusions of this article will be made available by the authors on request.

## Supplementary Materials

The following supporting information can be downloaded at: https://www.mdpi.com/article/10.3390/microorganisms12061207/s1.
