# Supplementary material for: Hepatitis B Virus Prevalence among HIV-Uninfected People Living in Rural and Peri-Urban Areas in Botswana
Source: Microorganisms. 2024 Jun 15;12(6):1207. doi: 10.3390/microorganisms12061207 (PMC11205512; doi:10.3390/microorganisms12061207)
Supplement: Supplementary file 1 [file microorganisms-12-01207-s001.zip › microorganisms-3024464-supplementary.pdf]

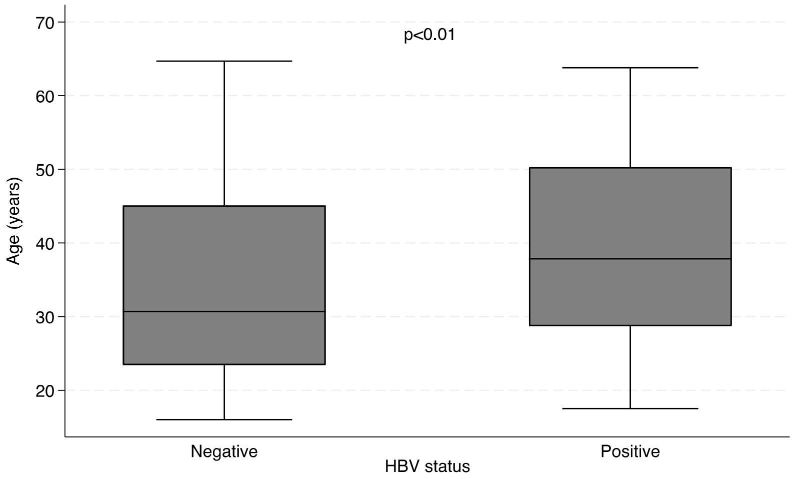

Supplemental Figure S1. Box plot showing the association between HBV status and age.

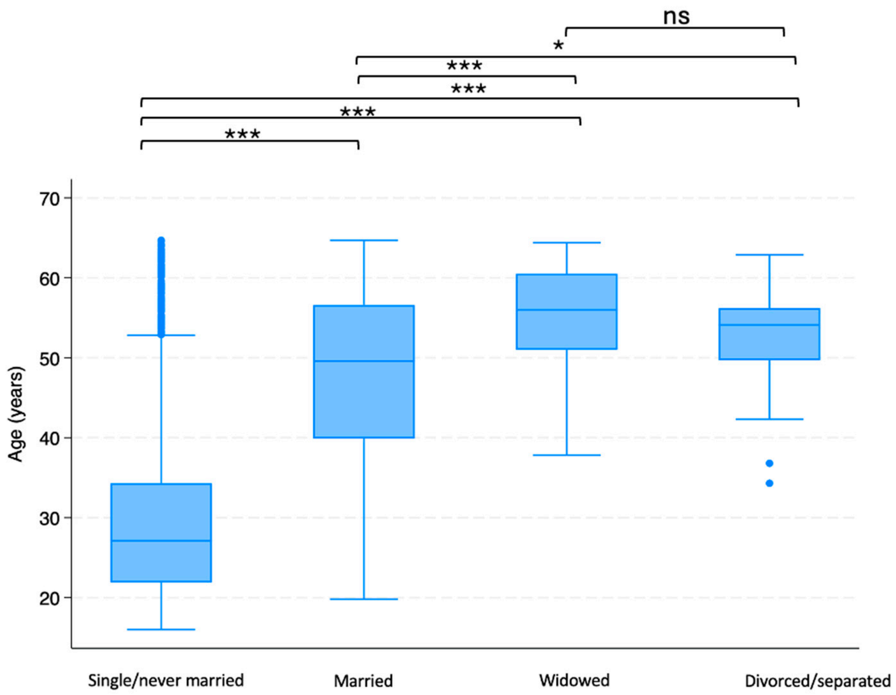

Supplemental Figure S2. Box plot showing the association between marital status and age (The significance levels of ns, *, **, *** and **** represent p-values > 0.05, <0.05, <0.01, <0.001 and <0.0001 respectively.).

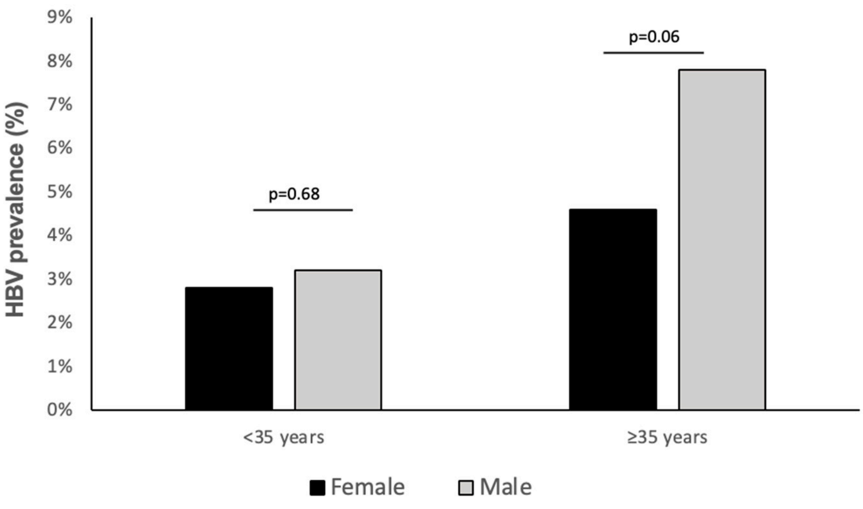

Supplemental Figure S3. HBV prevalence by age and sex.
